# Concordance between Histology, Immunohistochemistry, and RT-PCR in the Diagnosis of Feline Infectious Peritonitis

**DOI:** 10.3390/pathogens9100852

**Published:** 2020-10-18

**Authors:** Angelica Stranieri, Donatella Scavone, Saverio Paltrinieri, Alessia Giordano, Federico Bonsembiante, Silvia Ferro, Maria Elena Gelain, Sara Meazzi, Stefania Lauzi

**Affiliations:** 1 Department of Veterinary Medicine, University of Milan, 26900 Lodi, Italy; angelica.stranieri@unimi.it (A.S.); donatella.scavone@unimi.it (D.S.); alessia.giordano@unimi.it (A.G.); sara.meazzi@unimi.it (S.M.); stefania.lauzi@unimi.it (S.L.); 2 Veterinary Teaching Hospital, University of Milano, 26900 Lodi, Italy; 3 Department of Comparative Biomedicine and Food Science, University of Padua, 35020 Legnaro, Padova, Italy; federico.bonsembiante@unipd.it (F.B.); silvia.ferro@unipd.it (S.F.); mariaelena.gelain@unipd.it (M.E.G.); 4 Department of Animal Medicine, Productions and Health, University of Padua, 35020 Legnaro, Padova, Italy

**Keywords:** feline infectious peritonitis, FIP, feline coronavirus, immunohistochemistry, RT-PCR, agreement, diagnosis, cat

## Abstract

Histology, immunohistochemistry (IHC), and reverse transcription polymerase chain reaction (RT-PCR) have been used to diagnose feline infectious peritonitis (FIP), but no information regarding the comparison of their diagnostic performances on the same organ is available. The aims of this study were to determine the concordance among these tests and to evaluate which combination of tests and organs can be used in vivo. Histology, IHC, and nested RT-PCR (RT-nPCR) for feline coronavirus (FCoV) were performed on spleen, liver, mesenteric lymph node, kidney, large and small intestine, and lung from 14 FIP and 12 non-FIP cats. Sensitivity, specificity, predictive values, likelihood ratios, and concordance were calculated. IHC and RT-nPCR had the highest concordance in lung and liver, histology and IHC in the other organs. The sensitivity of histology, IHC, and RT-nPCR on the different organs ranged from 41.7 to 76.9%, 46.2 to 76.9%, and 64.3 to 85.7%, respectively, and their specificity ranged from 83.3 to 100.0%, 100% and 83.3 to 100.0%. Therefore, IHC is recommended when histology is consistent with FIP. If RT-nPCR is performed as the first diagnostic approach, results should always be confirmed with IHC. Lung or liver provide accurate information regardless of the method, while IHC is preferred to RT-nPCR to confirm FIP in the kidney or intestine.

## 1. Introduction

Feline coronavirus (FCoV) is a widespread, highly contagious virus belonging to the species *Alphacoronavirus 1* of the subgenus *Tegacovirus* (genus *Alphacoronavirus*, subfamily Orthocoronavirinae, family *Coronaviridae,* and order *Nidovirales*) [1]. FCoV may induce a transient or persistent enteric infection, characterized by mild or absent clinical signs, or an invariably lethal systemic disease called feline infectious peritonitis (FIP) [2,3,4]. According to their pathogenicity, FCoVs can be divided into two different biotypes: feline enteric coronavirus (FECV) and feline infectious peritonitis virus (FIPV), respectively [4]. It has been supposed that mutations of the viral genome could be responsible for the pathotypic switch, but they are more likely associated with a change in tropism from enterocytes to monocytes/macrophages, and consequently, with the systemic spread of the infection. FECV is, in fact, able to infect monocytes, albeit in a less efficient way compared to FIPV, and spread systemically [5,6]. Therefore, FECV is not necessarily confined to the intestinal tract, and the virus can also be detected in blood and other organs from healthy cats [7,8].

The pathogenesis of FIP is to date still not completely understood. FIPV can effectively and sustainably replicate in monocytes and macrophages, but other individual factors, such as the interaction with the immune response of the host, play a fundamental role in the onset and progression of the disease [3,9].

The absence of pathognomonic laboratory changes and clinical signs makes FIP a challenging disease to diagnose in vivo, especially when effusion is not present [10,11]. It is possible to achieve a diagnostic suspicion of FIP based on signalment, history, clinical signs, and laboratory analyses, but a definitive diagnosis relies on histopathological examination of tissues and FCoV detection by immunohistochemistry (IHC) [12]. The detection of histopathological changes typical of FIP in a cat with signalment, signs, and suggestive clinicopathological alterations can lead to the diagnosis of FIP with reasonable certainty [13]. When histological lesions are not strongly suggestive of FIP, the disease cannot be excluded, and immunostaining for FCoV antigen should be performed to confirm the diagnosis [14]. However, even if immunohistochemistry is empirically reported to be prone to false negative results for several reasons, no information about the sensitivity of this test is found in the literature [13,15]. Moreover, performing histopathology in vivo is a time-consuming procedure that requires the collection of surgical biopsy samples, which is often impossible if not discouraged in animals with suspected FIP [15,16].

Molecular tests provide more rapid results than histopathology and immunohistochemistry, but since no specific mutation has been found to distinguish the two pathotypes, reverse transcription polymerase chain reaction (RT-PCR) does not differentiate FECV from FIPV [6,17].

RT-PCR has been used in several studies to investigate the epidemiology of FCoVs, to assess the prevalence of FCoV serotypes, and to amplify and obtain viral RNA for sequencing and detecting mutations possibly related to virulence. Its diagnostic usefulness was evaluated on feces, effusion, blood, cerebrospinal fluid, aqueous humor, but the concordance between RT-PCR on tissues and IHC has not been evaluated yet using a standardized approach [12].

Hence, the first aim of this study was to evaluate the concordance between IHC and a reverse transcription nested PCR (RT-nPCR) in detecting FCoVs from tissues that might be collected in vivo. The second aim was to determine which organ and technique may be more useful to diagnose FIP, even when evident gross lesions are not detected. This study proves that it may be advisable to use IHC to both confirm and exclude the disease if histological lesions consistent with FIP are found. Conversely, RT-nPCR should not be used as a first diagnostic approach, i.e., on fine-needle aspiration biopsy (FNAB), due to its low specificity and sensitivity. Thus, it is always advisable to confirm RT-nPCR results with IHC. Based on concordance results, when multiple tissues cannot be analyzed, it would be preferable to examine either the lung or the liver, on which the probability to obtain accurate information is good, independent of the analytical method. On the contrary, when the kidney or intestine are sampled, IHC should be preferred to RT-nPCR as a confirmatory test since the concordance of the two methods is not sufficiently high, and the specificity is higher for IHC than for RT-nPCR.

## 2. Results

### 2.1. Final Diagnosis and Groups Formation

A total of 26 cats were included in the study. Specifically, 14 cats were allocated in the FIP group, and 12 in the non FIP group (Table 1 summarizes signalment, clinical suspicion, final diagnosis, and notes about tissue sampling of the cats included in the study).

Regarding the FIP group, five cats showed non-effusive forms of the disease, with primarily neurological (n°2, 3, 5) and ocular signs (n°1, 4), along with fever, lethargy, and anorexia. The remaining nine cats were affected by effusive FIP, with abdominal (n°6, 7, 8, 10, 12, 13, 14) and/or thoracic (n°9, 11, 12) effusion. When present, the effusion displayed features typical of FIP, such as yellowish color, viscous and sticky consistency, often associated with the presence of fibrin clots [18]. The exceptions were represented by cats n°12 and 14, whose effusions, collected in vivo, were initially consistent with FIP, while at necropsy had a purulent appearance, confirmed by cytologic evaluation, which allowed the categorization of the effusion as bacterial purulent effusion. 

Regarding the non FIP group, in four cases, FIP was considered as a possible differential diagnosis because of the presence of hyphemia (n°18), body cavity effusion and icterus (n°20), anisocoria and tremors (n°23), neurological signs and thoracic effusion (n°26). In eight cats, FIP was not clinically suspected, and death occurred for severe injuries (n°15, 19, and 22) or rodenticide poisoning (n°17) confirmed by necropsy, which revealed fractures and/or bleeding. The remaining cats were affected by inflammatory (n°16), infectious (n°21), and cardiac (n°24, 25) diseases. See Supplementary Table S1 for additional information regarding histologic lesions.

### 2.2. Histology

Histological examination was performed on 93 tissue samples obtained from FIP cats and 83 obtained from non FIP cats. Supplementary Tables S1 and S2 summarize histological findings recorded in each organ and results regarding macroscopic alterations; histology, IHC, and PCR recorded in all the tissues examined in this study, respectively. 

As reported in Table S2, histological lesions were also present in sections taken from organs not exhibiting evident macroscopic alterations. Overall, 55/93 tissues (59.1%) from FIP cats exhibited histological features consistent with the disease. Different types of lesions consistent with FIP were often simultaneously detected in the same animal and even within the same organ. Most of the cats (11/14, 78.6%) in the FIP group, in fact, showed lesions consistent with FIP in more than one organ, with different distribution based on the organ. Fibrinous serositis was consistently found, particularly on the spleen, liver, and lungs, while mesenteric lymph nodes were prevalently affected by granulomatous lesions, often with vasculitis. On the other hand, kidneys were often affected by lymphoplasmacytic infiltrates, in association either with granulomatous/pyogranulomatous lesions or vasculitis. Small and large intestine showed the various distribution of the above-mentioned lesions. In cat n°2, which presented neurological signs, histological lesions consistent with FIP were found only in the central nervous system (CNS) with the diagnosis of severe, chronic, multifocal to coalescent lymphoplasmacytic meningoencephalitis, and ependymitis. Not infrequently (38/93 tissues, 40.9%), the examined histological sections did not show either relevant lesions or lesions consistent with FIP. In the FIP group, the tissues that most often showed typical FIP histological lesions (Table 2) were the lung, kidney, and mesenteric lymph node, followed by the liver and spleen, while the small and large intestine were the organs less frequently affected by lesions imputable to FIP. 

As regards the non FIP group, the histological examination did not show lesions consistent with FIP in 77/83 samples (92.7%), and, therefore, lesions potentially consistent with FIP were found only in 6/83 tissues (7.2%), collected from 3/12 cats (25.0%). In this group, the organs that most frequently showed lesions possibly consistent with FIP were the kidney, followed by the lymph node, spleen, and liver. Lesions consistent with FIP were never detected in the small and large intestine (Table 2).

In a few cases, histological results were compatible but not highly suggestive for FIP and were categorized as negative or positive depending on findings in other tissues. In particular, 5/93 tissues from FIP cats showed lesions suspected for FIP, and three were categorized as negative (small intestine and lung of cat n°6 and large intestine of cat n°9) and two as positive (lymph node and kidney of cat n°10 and 11, respectively). In only 1/83 samples from non FIP cats, histology was non-conclusive, and the sample was categorized as positive considering findings in the other tissues (lung of cat n°25).

### 2.3. Immunohistochemistry

All the 12 cats (100.0%) assigned to the non FIP group tested negative at IHC for FCoV antigen in all the examined organs, with the exception of columnar enterocytes of cat n°21. This result was anyway considered negative because viral antigen was not found in the context of a histological lesion. On the other hand, 13/14 cats (92.8%) assigned to the FIP group tested positive in at least one of the examined organs, for a total of 53/93 immunohistochemically positive tissues (56.9%). The only cat in the FIP group with negative IHC in all the organs systematically sampled for this study was the cat n°2, in which lesions morphologically consistent with FIP and immunohistochemically positive were instead restricted on the CNS only. FCoV immunodetection was more frequent in lesions located in the lung and lymph node, followed by the spleen, liver, and kidney, while the small and large intestine were instead less often found to be positive (Table 2).

### 2.4. RT-nPCR

As regards the FIP group, all the cats showed a positive result in at least one of the examined tissues, and 70/92 samples (76.1%) gave a positive result, while the FCoV genome was not found in 22/92 tissues (23.9%). The organs which more frequently tested positive were the kidney and mesenteric lymph node, followed by spleen, lung, and large intestine, while the small intestine and liver were the least frequently positive organs. Considering the non FIP group, 5/12 cats (41.7%) exhibited a positive result in at least one tissue, and overall, 6/83 samples (7.2%) were positive, while FCoV RT-nPCR was negative in 77/83 samples (92.8%). The organs displaying the most frequent detection of viral RNA were the mesenteric lymph node and spleen. The kidney and small intestine were positive in only one case, while the large intestine, liver, and lung never tested positive (Table 2).

### 2.5. Concordance among Methods

The percentage of cases for which both compared methods classified the same sample as negative or positive, together with the corresponding Cohen’s kappa coefficient are reported in Table 3.

The highest rate of concordance was found between histology and IHC either on the whole set of tissues or each specific tissue. Exceptions were the lung, where the highest rate of concordance was found between IHC and RT-nPCR, and the liver, on which IHC and RT-nPCR had the same concordance of histology and IHC.

#### 2.5.1. Concordance between Histology and IHC

The tissue with the highest rate of concordance (close to or higher than 90.0%, *k* > 0.8) was the small intestine, followed by the liver, large intestine, and lymph node, while the kidney, spleen, and lung had the lowest concordance (80–90%, *k* coefficients between 0.6 and 0.8, i.e., strong). The highest rate of concordance was found in tissues with a high prevalence of double negative results. With regard to discordant results, the analysis of individual data (Table S2) revealed that the occurrence of negative IHC in tissues with lesions consistent with FIP was more frequent than positive IHC in tissues without lesions consistent with FIP. More specifically, IHC was negative in all the six samples from the non FIP group and 2/56 samples from the FIP group that were histologically consistent with FIP. Conversely, in the FIP group, IHC was positive in only 1/37 samples on which no lesions histologically consistent with FIP were found. In particular, the spleen of cat n°9 showed a moderate increase in the plasmacytic component and perivascular macrophages, not organized in a typical FIP lesion but immunohistochemically positive. 

#### 2.5.2. Concordance between Histology and RT-nPCR

As stated above, with the exception of the liver, that had an almost absolute concordance and a Cohen’s *k* coefficient that could be classified as “almost perfect”, in all the other organs, the percentage of concordance varied between 70 and 90% and the level of concordance measured by the *k* coefficient was strong (0.60–0.80) for the kidney and large and intestine, but moderate (0.40–0.60) for the lymph node, small intestine, and lung. With regard to discordant results, the analysis of individual data (Table S2) revealed that positive RT-nPCR in tissues without lesions potentially consistent with FIP occurred more frequently than negative RT-nPCR in tissues with lesions potentially consistent with FIP. In particular, RT-nPCR was negative in all the six samples from the non FIP group and in 1/56 samples from the FIP group that had lesions histologically consistent with FIP. Curiously, this latter sample was anyway positive in IHC. Conversely, RT-nPCR was positive in 15 tissues from cats with FIP and 6 tissues from the non FIP group in which no lesions consistent with FIP were found at histology. More specifically, 4/6 RT-nPCR-positive tissues from the non FIP group did not have histological lesions at all (spleen of cat n°20 and n°23, lymph node of cat n°21, kidney of cat n°19), while in 2/6 RT-nPCR-positive cases lymphoid hyperplasia was found (lymph node of cat n°20, small intestine of cat n°24). In the FIP group, no lesions at all were detected in six RT-nPCR-positive cases (spleen of cat n°3, kidney of cat n°12, small intestine of cats n°9 and 12, large intestine of cats n°2 and 12), while eight RT-nPCR-positive cases had lesions not consistent with FIP, such as lymphocytic hyperplasia (spleen of cat n°1, liver of cat n°14, lymph nodes of cats n°1, 2 and 14, large intestine of cat n°9), interstitial nephritis (cat n°13) and pulmonary emphysema (cat n°14).

#### 2.5.3. Concordance between IHC and RT-nPCR

The percentage of concordance was higher than 90%, and the level of concordance measured by the *k* coefficient was almost perfect only for the liver and lung. In all the other organs, the percentage of concordance varied between 80 and 90%, and the *k* coefficient was classified as strong (0.6–0.8). Overall, double negative results (negative results in both RT-nPCR and IHC) were more common than double positive results (positive result in both RT-nPCR and IHC). As regards discordant results, the analysis of individual data (Table S2) revealed that, with one single exception (lung of cat n°11) of positive IHC and negative RT-nPCR, in all the other discordant cases, RT-nPCR was positive in tissues with negative IHC. In particular, this occurred in the same 6 cases from the non FIP group and in 15/21 FIP tissues in which histology was classified as negative and RT-nPCR was positive (spleen of cats n°1 and 3, liver of cat n°14, lymph nodes of cats n°1, 2, and 14, kidney of cats n°5, 12, and 13, small intestine of cats n°9 and 12, large intestine of cats n°2, 9, and 12 and lung of cat n°14), whose histological findings have been described above. Additionally, IHC was negative, but RT-nPCR was positive in the kidney of cat n°4 and the large intestine of cat n°14, which, however, had histological lesions consistent with FIP.

### 2.6. Diagnostic Accuracy of Histology, Immunohistochemistry, and RT-nPCR

The diagnostic performance of histology, IHC, and RT-nPCR for the diagnosis of FIP on the different tissues analyzed in this study are reported in Table 4.

Histology showed absolute specificity and positive predictive value (PPV) only in the small and large intestine, while in all other organs except the kidney (83.3%), specificity was between 90.0 and 92.0%. The organs that less frequently showed lesions imputable to FIP, leading to very low sensitivity, were the small and large intestine with a sensitivity of 41.7 and 53.8%, respectively. The other organs also showed low sensitivity, except lung (76.9%), which also showed the likelihood ratios (LR)- result closest to 0.00 (i.e., the LR− that indicates a good probability to exclude the disease), followed by the kidney and mesenteric lymph node.

Immunohistochemistry showed absolute specificity in all tissues and, consequently, a high PPV, while its sensitivity, as well as the negative predictive value (NPV), were low, and the LR− was not close to 0.00. 

Conversely, specificity and PPV of RT-nPCR were absolute only for the liver, large intestine, and lung, although, for the kidney and small intestine, both the specificity and the PPV and especially the LR+ were high. However, sensitivity and the NPV of RT-nPCR, although not absolute and probably not relevant for diagnostic purposes, were higher than for IHC. In addition, in this case, however, the LR− was always distant from the optimal value of 0.00.

## 3. Discussion

This study was designed to achieve information about the concordance of different tests (histology, IHC, RT-nPCR) performed on the same organs of cats with FIP, aiming to find which one could be sampled in vivo to achieve an early diagnosis. Cats with both the non-effusive and effusive form were included for a fair comparison between methods applied to the same tissues. In fact, despite the clinical distinction, mixed or transition forms are common [9], and effusion and granulomatous lesions can be present to a greater or lesser degree and coexist [19]. Of course, in the effusive form, analysis of the effusion is very useful for diagnosis, even though the sensitivity of immunostaining is not absolute, and RT-nPCR lacks both sensitivity and specificity [20]. Therefore, tests on tissues might be necessary for diagnosis confirmation, even when effusion is present. The organ panel was selected with the major aim of including organs that can be sampled in vivo when trying to reach an early diagnosis in clinical practice. Organs reportedly affected by FIP lesions and, at the same time, accessible from a surgical point of view, were selected, aiming to guide the clinician to perform targeted surgical biopsies in cats suspected to have FIP. The analysis of every single test demonstrated, as expected that none of the tests have both absolute specificity and sensitivity. Histological lesions most frequently detected in the FIP group comply with the ones described in the literature as strongly consistent with FIP [9,21,22]. In some organs from FIP cats, histological lesions consistent with FIP were not detected. However, cats with FIP had histological lesions in more than one organ, except for two cats in which lesions were restricted to the kidney, which has been reported as one of the most frequently affected organs [9,18], and CNS. These findings suggest that the collection of biopsies from more than one organ might increase the probability of diagnosing FIP. However, histological lesions consistent with FIP, such as granulomatous inflammation and lymphoplasmacytic infiltrates [23], can also be detected in tissues from cats affected by diseases other than FIP. These lesions, however, in the absence of granulomatous lesions, cannot be considered absolutely diagnostic for FIP [21,24].

Differently from histology, IHC was positive only in cats with FIP. In non FIP cats, IHC positive signal was rarely found on villous superficial columnar epithelial cells of the large intestine. This may occur in non-symptomatic carriers since colonic enterocytes are thought to be the main site of viral persistence in the gut [7,25]. The few immunohistochemical positive results in the small and large intestine are quite surprising, given the tendency of FIP-related lesions to follow the course of the cranial mesenteric artery and their frequent involvement of the serosa of these organs [18]. However, IHC may also be negative in thoracic or abdominal organs when lesions are localized only in the CNS, as occurred in one cat of our caseload.

**Figure 1:**
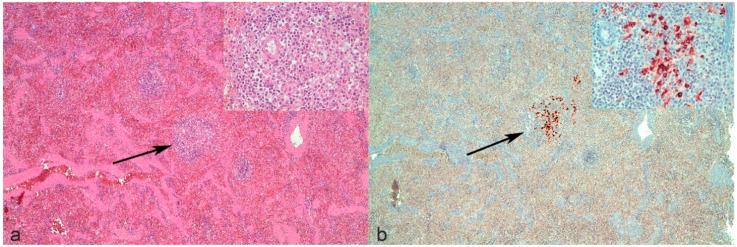
Spleen of cat n°9. (**a**) The parenchyma appeared quite normal at low magnification with only a few small periarteriolar lymphoplasmacytic infiltrates with macrophages, resembling normal white pulp (arrow). Inset: higher magnification of the infiltrate, no granulomas or vasculitis were clearly evident. (**b**) Immunolabeled macrophages were present inside the periarteriolar infiltrate (arrow). Inset: higher magnification.

It is noteworthy that revising the spleen of cat n°9 with a negative histological examination and positive IHC, only a mild increase in the perivascular number of macrophages was noticed multifocally, but was not considered as a typical lesion (Figure 1a). In light of the positivity to IHC (Figure 1b), these lesions could be considered as early lesions. This confirms that the presence of lesions potentially consistent but not absolutely diagnostic for FIP should induce the pathologist to perform IHC since the combination of tests may be more informative than histology alone.

Our results demonstrated that the number of RT-nPCR positivities exceeded those recorded in histology and IHC, due to the higher analytical sensitivity of this method [25] and to the possible occurrence of viremic episodes. Moreover, the viral burden in FCoV infected cats is reported to be very variable [26,27]. Therefore, some cats may have enough circulating viruses to be detected by molecular methods even when not affected by FIP.

The best concordance was found between histology and IHC, except for the lung, which showed the best concordance between IHC and RT-nPCR. In non FIP cats, the discordance between positive histology and negative IHC confirmed that lesions were due to causes other than FIP, as hypothesized above. In FIP cats, it may rather depend on the already reported variable distribution of lesions in different organs and viral antigens within lesions [21]. In these cases, however, other tissues from the same cat were positive at both histology and IHC, confirming that multiple tissues should be sampled to improve the probability to detect FIP lesions and/or positive IHC since IHC might not be confirmatory if only one section is analyzed [12]. A substantial limitation of this study is that the sampling of organs post-mortem does not exactly represent what can be sampled in a clinical setting, and our results cannot be directly transferred to bioptic samples collected in vivo. Moreover, tissues collected in vivo by bioptical means are reduced in size, and the possibilities of obtaining multiple sections from these kinds of samples, especially when they are sent to commercial laboratories, are low. Unfortunately, the same approach adopted in this study would not have been possible in live animals. The ultrasound-guided collection of bioptic samples immediately after euthanasia would be an alternative future approach to reproduce the clinical setting. However, results of histology, immunohistochemistry, and/or RT-nPCR may provide positive results even when evident gross lesions are not detected. Therefore, knowing which tissues might be preferable to collect, even if not affected by macroscopic or sufficiently large lesions to be detected by imaging means, could possibly be useful.

The low concordance between RT-nPCR and histology or IHC was generally due to positive RT-nPCR in tissues that were negative for histology and/or IHC. This is likely due to the above mentioned high analytical sensitivity of RT-nPCR, coupled with the possible systemic spread of FCoV in infected cats not affected by FIP [6,17,28]. The single exception was represented by one lung sample from an FIP cat, which had positive IHC but negative RT-nPCR. Different hypotheses could explain this discrepancy. First, lesions could be restricted to sections processed for IHC and not in those intended for RT-nPCR, despite collected tissue sections always being adjacent to each other. Second, the amount of viral RNA in the sample intended for RT-nPCR might not have been adequate to produce enough amplification products. Third, the FCoV genome within the lesion could have undergone mutation(s) that did not allow the binding of primers to target sequences. This latter hypothesis is unlikely, either because the 3′-UTR’s target of the RT-nPCR is well conserved in FCoVs [28] or because the kidney from the same cat was RT-nPCR positive, although simultaneous infection by different mutated strains cannot be excluded.

It is noteworthy that in non FIP cats, positive results of RT-nPCR may be misleading. Positive results were found, especially in the mesenteric lymph node and spleen. These latter organs are thought to harbor high viral loads because of the migration of FECV infected monocytes through the vascular network [27]. Conversely, the absence of RT-nPCR positive results in the liver and lung of non FIP cats is partly surprising and in disagreement with those studies which identified these organs as probable sites of FCoV persistence in healthy cats, as the results recorded in the large intestine. Again, this finding could be imputable to the low sample size of non FIP cats [25]. However, in both FIP and non FIP cats, the rate of concordance between RT-nPCR and IHC was very variable in the different tissues. Again, this indicates that the probability of diagnosing FIP, independent of the method used, increases when several organs are sampled. Alternatively, it may be advisable to sample the liver, lung, and small intestine since these organs had the highest rate of positive results and may provide the same information independent of the diagnostic method used.

Indeed, the choice of tissue and test that more likely might provide diagnostic information should also be based on information about diagnostic accuracy. From this perspective, our results confirmed that, regardless of the examined organ, positive IHC is always diagnostic for FIP since the specificity and positive predictive value reached 100%. On the contrary, negative IHC does not allow the exclusion of FIP, based on the low sensitivity and negative predictive value and on the high negative LR. These results, in fact, indicate that, in the case of negative IHC, the chance that the cat is affected by FIP is not null [29]. Nevertheless, it should again be considered that performing IHC on a wide range of organs and lesions increases the probabilities to diagnose FIP. Additionally, considering results from CNS tissues from cat n°2, which were not included in the calculation of diagnostic performance of IHC, the diagnostic accuracy of IHC would have reached 100%. This additional information confirms that IHC performed on multiple tissues collected at necropsy, including CNS, may be absolutely diagnostic for FIP. However, this information would also deviate from the aims of this study, which is intended to recommend organs to sample in clinical practice on live animals, based on the frequency of positive results. From this standpoint, the higher specificity of IHC compared with histology, coupled with the low sensitivity of histology, suggest that a negative histological result alone cannot rule out FIP and that in case of strong clinical suspicion, it is advisable to obtain serial sections of the same tissue to perform IHC, to increase the probability of finding the FCoV antigen within histological lesions, which may not have been included in the first slide.

Contrary to IHC, RT-nPCR showed an overall higher sensitivity but lower specificity, as already reported [17], with rare exceptions. According to our results, positive RT-nPCR cannot be considered diagnostic for FIP, except for the liver, large intestine, and lung, in which this method exhibited absolute specificity. Conversely, diagnostic accuracy on the kidney proved to be the highest, as demonstrated by the high positive likelihood ratio, that unlike predictive values, was not affected by the prevalence of the disease. In some cases, the sensitivity of RT-nPCR was low, especially in the liver and large intestine. Results regarding RT-nPCR on the large intestine were quite surprising since samples from this latter organ are more likely expected to give false positive RT-nPCR results due to the presence of FCoV in the gastrointestinal tract of shedder cats. A possible explanation for this finding could be the action of fecal RNAse on nucleic acids and their degradation. Moreover, the high rate of negative RT-nPCR results recorded in this study in the small and large intestine could depend on a decreased viral fecal excretion, which has been demonstrated to occur in FIP affected cats, hypothetically due to the impaired tropism of mutated FCoV for intestinal epithelial cells [30,31]. On the other hand, non FIP cats are less likely to shed the virus in the feces compared with FIP cats [17]. Nevertheless, the small sample size of the non FIP group could also have affected the results, and also performing the RT-nPCR on feces would have been useful to confirm if the cats were not shedding FCoV at the moment of sampling. Specificity was notably low in the mesenteric lymph node and spleen, suggesting that an RT-nPCR positivity in these organs, which are frequently sampled in clinical routine for diagnostic purposes, cannot be considered as diagnostic for FIP. This seems to be in contrast with the high sensitivity and specificity of quantitative RT-nPCR on the mesenteric lymph node that has been recently reported [15]. Overall, the low specificity of RT-nPCR makes it mandatory to perform histology and IHC independently of the RT-nPCR results, except in the liver and lung, where a positive result is absolutely specific for FIP.

## 4. Materials and Methods

### 4.1. Caseload

Tissue samples were collected post-mortem at the Veterinary Teaching Hospital of Milan from cats affected by FIP or other systemic diseases or serious injuries, deceased or euthanized, and subjected to necropsy for diagnostic purposes. All the above methods were performed within routine diagnostic procedures and with the owner’s written informed consent about the use of tissues and samples for research. Therefore, according to the Ethical Committee of the University of Milan (decision n° 2, 2016), residual aliquots of tissues were used for research purposes without any additional formal request of authorization to the Ethical Committee.

The inclusion criteria were:

- The possibility to collect and process tissues within six hours after death;
- The availability of signalment, history, and clinical information (physical examination, clinical pathology, diagnostic imaging, depending on the clinical presentation). This information, along with necropsy findings and histology results, was used to classify the cats in the FIP or non FIP group [32].

### 4.2. Sample Collection and Processing

Samples were collected from the spleen, liver, mesenteric lymph node, kidney, large intestine, small intestine, and lung of the necropsied cats, paying particular attention to sample tissues with gross lesions, whenever present, and to always include the organs serosa (see Table 1 for a detailed list of sampled organs).

When possible, data regarding evident macroscopic alterations frequently seen in FIP, namely: thickening of the serosa, focal or diffuse nodular lesions, organs increase in size, were recorded (see Supplementary Table S2). Samples were then processed as described below.

For diagnostic purposes, tissues from other organs showing macroscopic alterations, as well as organs not visibly affected but plausibly involved based on the patient’s history (e.g., brain and cerebellum from cats with neurological signs), were also sampled during a necropsy to perform routine histology and immunohistochemistry. Since RT-nPCR was not performed on these tissues, the corresponding results were not included in comparison with RT-nPCR but were used to achieve accurate classification of cases in the FIP or non FIP group as detailed below.

From each organ, a sample was taken and sectioned with a sterile scalpel in two immediately adjacent halves of approximately one-centimeter diameter each. If evident nodular lesions were present, the two halves were obtained, sectioning the sample in the center of the lesion. One half was placed in plain tubes and immediately frozen at −20 °C for molecular biology, while the other half was collected into 10% neutral-buffered formalin to perform histology and immunohistochemistry.

### 4.3. Histology and Immunohistochemistry

Histopathology was performed by an experienced veterinary pathologist, blinded to both gross lesions as well as to the patient’s signalment and history. Formalin-fixed samples were sent to the Department of Comparative Biomedicine and Food Science of the University of Padua. Sections (4 μm) obtained from formalin-fixed paraffin-embedded samples were stained with hematoxylin–eosin for histology with an automated stainer (Autostainer XL, Leica Biosystems, Wetzlar, Germany). For immunohistochemistry (IHC), 4 μm paraffin sections were placed on surface-coated slides (Superfrost Plus, ThermoFisher Scientific, Milan, Italy). Immunostaining was performed with an automatic immunostainer (Ventana Benchmark XT, Ventana Medical System, Tucson, AZ, USA). Antigen retrieval was performed with standard CC1 (Heat Induced Epitope Retrieval, HIER, in Tris-EDTA buffer pH 7 at 95 °C for 44 min). As the primary antibody, a mouse monoclonal antibody against the feline coronavirus was used (dilution 1:500, clone FIPV3-70 Serotec, Oxford, UK). After incubation at 37 °C for 30 min, a kit with a secondary antibody with horseradish peroxidase (HRP)-conjugated polymer that binds to mouse and rabbit primary antibodies (ultraViews Universal DAB, Ventana Medical System, Tucson, AZ, USA) was used. All reagents were dispensed automatically except for the primary antibody, which was manually dispensed. FIP positive tissues were used as internal positive controls. For negative controls, the antibody diluent (Ventana Medical Systems) was applied instead of the primary monoclonal antibodies.

### 4.4. FCoV RT-nPCR

From frozen–thawed samples, RNA was obtained using a NucleoSpin RNA kit (Macherey-Nagel, Bethlehem, PA, USA). Twenty milligrams of tissue were thinly shredded on sterile plates using sterile scalpels and vigorously vortexed in RA1 lysis buffer until completely dissolved. All the further steps were performed according to the manufacturer’s instructions. RNA samples were then frozen at −80 °C or immediately used for RT-nPCR. RT-nPCR targeting a 177 bp product of the highly conserved 3′ untranslated region (3′ UTR) of the genome of both type I and type II FCoV was used [28]. RNA pre-analytical quality control targeting vertebrate 12S rRNA locus [33] was performed on randomly selected samples (results not shown). FCoV RT-nPCR positive RNA was used as positive control and a FIP-negative sample as negative control. RT-nPCR products were visualized under UV transilluminator on a 1.5% agarose gel stained with ethidium bromide.

### 4.5. Formation of Groups

Cats included in this study were divided into two groups based on the following criteria.

- FIP group: cats suspected to have FIP based on history and/or clinical, hematological, biochemical, and serum protein electrophoretic alterations [11] on which diseases other than FIP were excluded, showing gross and histological lesions consistent with FIP in at least one tissue, as reported below, including tissues sampled for diagnostic purposes and not included in this study (e.g., brain or cerebellum).
- Non FIP group: cats with or without a clinical presentation potentially consistent with FIP, on which diseases other than FIP were already diagnosed on a clinical basis (e.g., based on results of diagnostic imaging or cytology) and/or without any histological lesion consistent with FIP.

### 4.6. Analysis of Results

**Figure 2:**
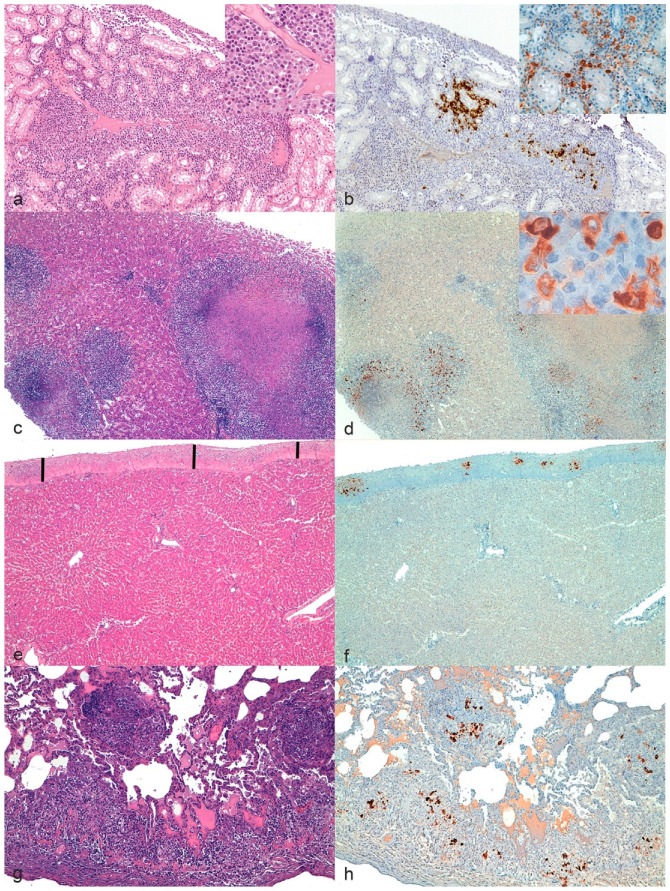
Feline infectious peritonitis (FIP), cat. Typical lesions. (**a**,**c**,**e**,**g**) hematoxylin–eosin; (**b**,**d**,**f**,**h**) immunohistochemistry for feline coronavirus. (**a**,**b**) Chronic interstitial nephritis, kidney. (**a**) A moderate perivascular lymphoplasmacytic and macrophagic infiltrate were present. (**b**) Distribution of immunolabeled cells. Inset: higher magnification. (**c**,**d**) Granulomatous hepatitis, liver. (**c**) Multifocal nodular infiltrates with central necrosis. (**d**) Distribution of the immunolabeled cells in the infiltrates. Inset: High magnification of macrophages with intense cytoplasmic immunolabeling. (**e**,**f**) Fibrinous perihepatitis, liver. (**e**) The black vertical bars indicate the thick band of fibrin along the serosal surface with few aggregates of inflammatory cells. (**f**) Multifocal distribution of the immunolabeled cells. (**g**,**h**) Lung. Diffuse and multifocal interstitial infiltrates (**g**) associated with immunolabeled cells (**h**).

For each organ, lesions were considered consistent with FIP if showing one or more of the following typical FIP patterns (Figure 2) [9,18]:

- Pyogranulomas on one or more serosal surfaces;
- Granulomas with or without necrotic areas;
- Lymphocytic and plasmacytic infiltrates in specific sites (e.g., band-like infiltrate in serosal surfaces, perivascular infiltrate in meninges and CNS);
- Granulomatous to necrotizing vasculitis and fibrinous serositis.

Conversely, histological lesions were considered non-consistent with FIP if changes consistent only with diseases other than FIP or no lesions at all were present.

Histological lesions not diagnostic for FIP as a standalone, but consistent with FIP if associated with signalment, history, gross lesions, and other histological lesions (e.g., pyogranulomas) suggestive of FIP, were considered potentially consistent with FIP. These lesions (e.g., multifocal perivascular increase in macrophages) were categorized as positive or negative depending on the lesions recorded in other organs, as it would happen in a clinical setting.

**Figure 3:**
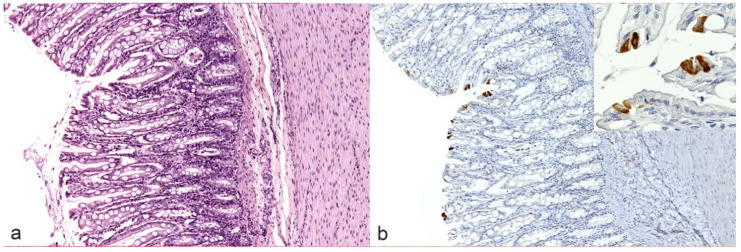
Large intestine. No lesions are detectable in the mucosa (**a**), but few superficial columnar epithelial cells show cytoplasmic immunolabeling without associated infiltrates (**b**). Inset: higher magnification.

As previously described by Pedersen and Kipar, immunohistochemistry was considered positive if FCoV antigen was detected within typical histological lesions above described [9,18]. On the contrary, according to Kipar and colleagues, the detection of FCoV antigen in few scattered tissue macrophages (e.g., Kupffer cells in the liver, pulmonary interstitial macrophages in the lungs as well as individual sinus macrophages in the lymph nodes) or epithelial cells (e.g., intestinal columnar cells, likely infected by FECV, Figure 3) only, was not considered as a positive result [25].

RT-nPCR was considered positive if it showed a 177bp band on agarose gel electrophoresis [28].

For histology, IHC and RT-nPCR, true-positive (results consistent with FIP in cats with FIP) and false-positive results (results consistent with FIP in cats without FIP), as well as true-negative (results not consistent with FIP in cats without FIP) and false-negative results (results not consistent with FIP in cats with FIP) were recorded.

Sensitivity, specificity, accuracy, positive and negative predictive values, as well as positive and negative likelihood ratios (LR+ and LR−, respectively) were then calculated for histology, IHC, and RT-nPCR.

The concordance between histology, IHC and RT-nPCR was assessed using Cohen’s *k* coefficient. Concordance was classified as absent (*k* < 0); minimal (0 < *k* < 0.20); weak (0.21 < *k* < 0.40); moderate (0.41 < *k* < 0.60); strong (0.61 < *k* < 0.80), and almost perfect (0.81 < *k* < 1) [34].

## 5. Conclusions

In conclusion, results from this study prove that the frequency of positive results obtained with different methods (histology, IHC, and RT-nPCR) on FIP cats varies depending on the analyzed tissue. Even if with a low frequency, some histological results consistent with FIP were also recorded in non FIP cats. Conversely, IHC had a 100% specificity, and, therefore, it may be advisable to use IHC to confirm and exclude the disease in the presence of histological lesions consistent with FIP. On the other hand, RT-nPCR is not recommended as a first diagnostic approach, i.e., on fine-needle aspiration biopsy (FNAB), due to its lower specificity and sensitivity. Therefore, it is always advisable to confirm possible positive or negative RT-nPCR results with IHC. However, data concerning concordance suggest that, when both tests cannot be performed, it would be preferable to examine either the lung or liver, on which the probability to obtain accurate information, independent of the analytical method, is good. Instead, when the kidney or intestine are sampled, IHC should be preferred to RT-nPCR as a confirmatory test since the concordance of the two methods is not sufficiently high, and the specificity is higher for IHC than for RT-nPCR.

## Figures and Tables

**Table 1:** List of the cats included in the study, with signalment, clinical suspicion, final diagnosis, and tissues not collected.

	N°	Breed	Age	Sex	Clinical Suspicion	Final Diagnosis
**FIP**	1	DSH	8 m	F	FIP	Non-effusive FIP ^a^
	2	DSH	6 m	M	FIP	Non-effusive FIP
	3	DSH	10 m	FS	FIP	Non-effusive FIP ^b^
	4	DSH	1.5 y	FS	FIP	Non-effusive FIP
	5	DSH	n/d	F	FIP/Neoplasia	Non-effusive FIP
	6	MC	9 y	MN	FIP	Effusive FIP
	7	ES	6 m	M	FIP	Effusive FIP
	8	DSH	5 y	FS	FIP	Effusive FIP ^c^
	9	R	1 y	MN	FIP	Effusive FIP
	10	DSH	1 y	FS	FIP	Effusive FIP
	11	DSH	1 y	MN	FIP	Effusive FIP
	12	DSH	2 y	MN	FIP/Purulent septic effusion	Effusive FIP ^d^
	13	DSH	n/d	MN	FIP	Effusive FIP
	14	MC	1 y	F	FIP/Septic	Effusive FIP
**Non FIP**	15	DSH	15 y	F	Trauma	Bite wounds
	16	DSH	15 y	FS	Neoplasia	Chronic pneumonia
	17	DSH	1.5 m	FS	Poisoning	Rodenticide poisoning
	18	DSH	5 y	F	FIP	Diabetes mellitus ^e^
	19	DSH	n/d	M	Trauma	Multiple wounds
	20	DSH	n/d	MN	FIP/Neoplasia	Pulmonary adenocarcinoma
	21	DSH	2 m	F	FPV infection	FPV infection
	22	DSH	n/d	F	Trauma	Multiple wounds
	23	DSH	2 y	M	FIP	Heart failure
	24	DSH	1.5 y	FS	Heart failure	Heart failure
	25	DSH	7 y	MN	Heart failure	Heart failure
	26	DSH	9 y	MN	FIP/Neoplasia	Thymic carcinoma

Abbreviations: d, days; DSH, domestic shorthair; ES, Exotic shorthair; F, female; FPV, feline panleukopenia virus; FS, female spayed; m, months; M, male; MC, Maine Coon; MN, male neutered; R, Ragdoll; y, years; n/d, not determined. ^a^ lung not collected; ^b^ small intestine not collected; ^c^ small and large intestine not collected; ^d^ mesenteric lymph node not collected; ^e^ mesenteric lymph node not collected.

**Table 2:** Positive results at histology, immunohistochemistry, and nested reverse transcription polymerase chain reaction RT-nPCR for each tissue collected from the feline infectious peritonitis (FIP) and non FIP groups.

	FIP			Non FIP		
	Histology	IHC	RT-nPCR	Histology	IHC	RT-nPCR
**Spleen**	8/14 (57.1%)	8/14 (57.1%)	10/13 (76.9%)	1/12 (8.3%)	0/12 (0.0%)	2/12 (16.7%)
**Liver**	8/14 (57.1%)	8/14 (57.1%)	9/14 (64.3%)	1/12 (8.3%)	0/12 (0.0%)	0/0 (0.0%)
**Lymph node**	8/13 (61.5%)	8/13 (61.5%)	11/13 (84.6%)	1/11 (9.1%)	0/11 (0.0%)	2/11 (18.2%)
**Kidney**	9/14 (64.3%)	8/14 (57.1%)	12/14 (85.7%)	2/12 (16.7%)	0/12 (0.0%)	1/12 (8.3%)
**Small intestine**	5/12 (41.7%)	5/12 (41.7%)	8/12 (66.7%)	0/12 (0.0%)	0/12 (0.0%)	1/12 (8.3%)
**Large intestine**	7/13 (53.8%)	6/13 (46.2%)	10/13 (76.9%)	0/12 (0.0%)	0/12 (0.0%)	0/12 (0.0%)
**Lung**	10/13 (76.9%)	10/13 (76.9%)	10/13 (76.9%)	1/12 (8.3%)	0/12 (0.0%)	0/12 (0.0%)

For each test, positive results are expressed both in numbers and percentages. Abbreviations: IHC, immunohistochemistry.

**Table 3:** Percentage of concordance and Cohen’s *k* coefficient (95% confidence interval between parentheses), for all tissues considered as a whole and for every single tissue.

	Histology vs. IHC	Histology vs. RT-nPCR	IHC vs. RT-nPCR
**All tissues**	92.0%	81.6%	86.9%
	0.82 (0.73–0.91)	0.62 (0.50–0.73)	0.72 (0.62–0.83)
**Spleen**	88.5%	76.0%	84.0%
	0.74 (0.46–1.00)	0.51 (0.19–0.83)	0.68 (0.40–0.95)
**Liver**	96.2%	92.3%	96.2%
	0.91 (0.75–1.00)	0.83 (0.60–1.00)	0.91 (0.75–1.00)
**Lymph node**	91.7%	75.5%	83.3%
	0.82 (0.59–1.00)	0.51 (0.19–0.83)	0.67 (0.40–0.95)
**Kidney**	88.5%	87.5%	80.8%
	0.75 (0.50–1.00)	0.71 (0.42–1.00)	0.62 (0.34–0.90)
**Small intestine**	100%	75%	75%
	1.00 (1.00–1.00)	0.52 (0.11–0.93)	0.52 (0.11–1.00)
**Large intestine**	95.8%	87.5%	84.0%
	0.89 (0.69–1.00)	0.73 (0.46–1.00)	0.64 (0.34–0.94)
**Lung**	84.0%	76.0%	92.0%
	0.67 (0.37–0.97)	0.50 (0.15–0.85)	0.83 (0.61–1.00)

**Table 4:** Diagnostic performances of histology, immunohistochemistry, and RT-nPCR for the diagnosis of FIP on each kind of tissue analyzed in the study.

	Histology						IHC						RT-nPCR					
	Spec	Sens	PPV	NPV	LR+	LR−	Spec	Sens	PPV	NPV	LR+	LR−	Spec	Sens	PPV	NPV	LR+	LR−
Spleen	91.7	57.1	88.9	64.7	6.85	0.46	100	57.1	100	66.7	n/a	0.43	83.3	76.9	83.3	76.9	4.62	0.28
Liver	91.7	57.1	88.9	64.7	6.85	0.46	100	57.1	100	66.7	n/a	0.43	100	64.3	100	70.6	n/a	0.36
LN	90.9	61.5	88.9	66.7	6.76	0.42	100	61.5	100	68.8	n/a	0.38	81.8	84.6	84.6	81.8	4.65	0.19
Kidney	83.3	64.3	81.8	66.7	3.85	0.42	100	57.1	100	66.7	n/a	0.43	91.7	85.7	92.3	84.6	10.3	0.16
SI	100	41.7	100	66.7	n/a	0.58	100	50.0	100	66.7	n/a	0.58	91.7	66.7	88.9	73.3	8.00	0.36
LI	100	53.8	100	66.7	n/a	0.46	100	46.2	100	63.2	n/a	0.54	100	76.9	100	80.0	n/a	0.23
Lung	91.7	76.9	90.9	78.6	9.2	0.25	100	76.9	100	80.0	n/a	0.23	100	76.9	100	80.0	n/a	0.23

Abbreviations: LI, large intestine; LN, mesenteric lymph node; LR+, positive likelihood ratio; LR−, negative likelihood; n/a, not applicable; NPV, negative predictive value; PPV, positive predictive value; Sens, sensitivity; SI, small intestine; Spec, specificity.

## Supplementary Materials

The following are available online at https://www.mdpi.com/2076-0817/9/10/852/s1, Supplementary Table S1. Details on histological lesions recorded for each sample included in the study. Abbreviations. Eo, eosinophilic, F, fibrinous; G, granulomatous; LP, lymphoplasmacytic; N, neutrophilic; M, macrophagic; PV, perivascular. Lesions highly suggestive and classified as consistent with FIP are reported in Italic. ^a^ Lesions compatible with FIP, classified as negative (not consistent with FIP). ^b^ Lesions compatible with FIP, later on, classified as positive (consistent with FIP). Supplementary Table S2. Results of macroscopic and histological examination, immunohistochemistry, and RT-nPCR obtained on each collected sample. Negative (not consistent with FIP) and positive (highly suggestive, therefore compatible with FIP) results are listed. Abbreviations: N, negative; n/a, not applicable (sample not collected); n/d, not done; P: positive.
